# New Viral Diseases and New Possible Remedies by Means of the Pharmacology of the Renin-Angiotensin System

**DOI:** 10.1155/2023/3362391

**Published:** 2023-07-12

**Authors:** Giovanni Sansoè, Manuela Aragno

**Affiliations:** ^1^Gastroenterology Unit, Humanitas Institute, Gradenigo Hospital, Corso Regina Margherita 10, 10153 Torino, Italy; ^2^Department of Clinical and Biological Sciences, University of Turin, Turin, Italy

## Abstract

All strains of SARS-CoV-2, as well as previously described SARS-CoV and MERS-CoV, bind to ACE2, the cell membrane receptor of *β*-coronaviruses. Monocarboxypeptidase ACE2 activity stops upon viral entry into cells, leading to inadequate tissue production of angiotensin 1-7 (Ang1-7). Acute lung injury due to the human respiratory syncytial virus (hRSV) or avian influenza A H7N9 and H5N1 viruses is also characterized by significant downregulation of lung ACE2 and increased systemic levels of angiotensin II (Ang II). Restoration of Ang1-7 anti-inflammatory, antifibrotic, vasodilating, and natriuretic properties was attempted at least in some COVID-19 patients through i.v. infusion of recombinant human ACE2 or intranasal administration of the modified ACE2 protein, with inconsistent clinical results. Conversely, use of ACE inhibitors (ACEis), which increase ACE2 cell expression, seemed to improve the prognosis of hypertensive patients with COVID-19. To restore Ang1-7 tissue levels in all these viral diseases and avoid the untoward effects frequently seen with ACE2 systemic administration, a different strategy may be hypothesized. Experimentally, when metallopeptidase inhibitors block ACE2, neprilysin (NEP), highly expressed in higher and lower airways, starts cleaving angiotensin I (Ang I) into Ang1-7. We suggest a discerning use of ACEis in normohypertensive patients with *β*-coronavirus disease as well as in atypical pneumonia caused by avian influenza viruses or hRSV to block the main ACE-dependent effects: Ang II synthesis and Ang1-7 degradation into angiotensin 1-5. At the same time, i.v.-infused Ang I, which is not hypertensive provided ACE is inhibited, may become the primary substrate for local Ang1-7 synthesis via ubiquitous NEP; i.e., NEP could replace inadequate ACE2 function if Ang I was freely available. Moreover, inhibitors of chymase, a serine endopeptidase responsible for 80% of Ang II-forming activity in tissues and vessel walls, could protect patients with atypical pneumonia from Ang II-mediated microvascular damage without reducing arterial blood pressure.

## 1. Introduction

Three species of genus *β*-coronavirus (SARS-CoV, MERS-CoV, and SARS-CoV-2) have caused atypical pneumonia in humans, called severe acute respiratory syndrome (SARS), Middle East respiratory syndrome (MERS), and coronavirus disease of 2019 (COVID-19), respectively. The SARS outbreak first occurred in China in 2002 and the MERS outbreak in Saudi Arabia in 2012. They led to hundreds of deaths with a fatality rate of 10% and 37%, respectively [1]. The severe acute respiratory syndrome coronavirus 2 (SARS-CoV-2) infection arrived on the medical scene in December 2019, and in less than 3 months, it was declared a pandemic. To date, COVID-19 has been diagnosed in more than 760 million people worldwide, and more than 6.8 million patients have died from the illness.

Approved vaccines against SARS-CoV-2 are being administered, but a significant number of infections still occur in people who are not vaccinated or, most dramatically, because of the progressive appearance of immune-evasive viral variants often characterized by increased infectivity but seldom by increased morbidity and mortality rates [2, 3]. Immunocompromised patients, who may not be able to mount an appropriate response to vaccines, have monoclonal antibodies as the only prophylactic agents, which may not work because of the propensity of the viral spike to evolve and escape neutralization [4, 5]. When it comes to the treatment of COVID-19, besides necessary ventilatory support, dexamethasone, remdesivir, molnupiravir, nirmatrelvir, and monoclonal antibodies, such as the REGN-CoV-2 cocktail, do not significantly improve the survival rate of severe patients with acute respiratory distress syndrome (ARDS) caused by SARS-CoV-2 [6–8]. Nonetheless, remdesivir treatment within two days of admission, with or without dexamethasone, reduces in-hospital mortality due to COVID-19, at least among elderly patients receiving supplemental oxygen on the day of admission [9, 10].

It is well known that an exuberant cytokine release and the consequential inappropriate hyperinflammatory reaction fuel the most severe cases of COVID-19 [11]. A dramatic consequence of this “cytokine storm” is invariably acute lung injury (ALI), clinically characterized by ARDS with exponentially elevated systemic levels of tumour necrosis factor-*α* (TNF-*α*) and interleukin-1 (IL-1), along with a systemic inflammatory response characterized by hypotension, organ hypoperfusion, fever with increased heart rate, and altered mental status [12, 13].

**Figure 1:**
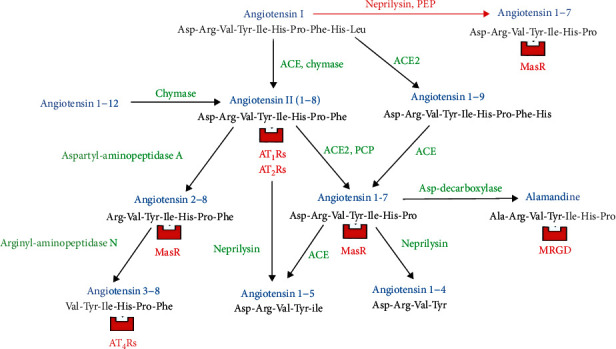
Diagram depicting pathways of synthesis and degradation of angiotensins in classical and nonclassical local/tissue RAS, with respective receptors for each bioactive peptide. ACE: angiotensin-converting enzyme; ACE2: angiotensin-converting enzyme type 2; AT_1-2-4_Rs: angiotensin type 1-2-4 receptors; MasR: Mas receptor; MRGD: Mas-related G protein-coupled receptor member D; PCP: prolyl carboxypeptidase; PEP: prolyl endopeptidase. The main degradative pathway for Ang II in normal humans is through the sequential actions of plasma aminopeptidases A and N, not through ACE2; serine endopeptidase chymase, in the heart, renal tubules, and ubiquitous mast cells, converts Ang I into Ang II as efficiently as ACE does in the vascular endothelium; Zn-metalloendopeptidase neprilysin cleaves angiotensin I into Ang1-7. Neprilysin, based on the occasional substrate available, generates Ang1-7 from Ang I but may metabolize Ang1-7 to form Ang1-4.

All genus *β*-coronaviruses so far described use the metallopeptidase angiotensin-converting enzyme type 2 (ACE2) as the cell membrane receptor to enter human cells [14, 15], a process that leads to annihilation of the key functions of ACE2: control of hemodynamics and systemic inflammation. ACE2 functionally belongs to the so-called nonclassical local/tissue renin-angiotensin system (RAS), where it is primarily involved in the conversion of angiotensin II (Ang II) into angiotensin 1-7 (Ang1-7). Nonclassical RAS itself (Figure 1) is an extremely complex and adaptable network of enzymes and active peptides that is involved in the regulation of extracellular fluid volume, arterial pressure, tissue blood perfusion, and inflammation.

It is to be remembered also that human respiratory syncytial virus (hRSV) and avian influenza A H7N9 and H5N1 viruses may cause ALI characterized by significant downregulation of lung ACE2 and increased systemic levels of Ang II [16–18]. As for coronaviruses, it is worth mentioning that another component of nonclassical RAS (i.e., aminopeptidase N, in part responsible for Ang II catabolism) (Figure 1) is the human cell membrane receptor of coronavirus hCoV-229E, which circulates worldwide and causes just mild respiratory disease (i.e., the common cold) [19].

For these reasons, maybe the most sensible clinical answer to all these viral illnesses, which have much in common, i.e., considerable damage to tissue ACE2 activity, should be sought in the intricate links between RAS malfunction and inflammatory cytokine release.

## 2. Classical and Nonclassical RAS (Figure 1)

The classical RAS is controlled by renin, which cleaves the decapeptide angiotensin I (Ang I) from plasma angiotensinogen. In turn, angiotensin-converting enzyme (ACE) converts Ang I into Ang II, the octapeptide that essentially stimulates angiotensin type 1 receptors (AT1Rs), leading to increased cardiac inotropism, arterial vasoconstriction, catecholamine release, aldosterone secretion, and renal sodium retention (the so-called ACE-Ang II-AT1R pathway) [20].

The nonclassical RAS is a further network of enzymes and angiotensins derived from Ang II. ACE2 is a transmembrane protein with an extracellular N-terminal domain, which contains a monocarboxypeptidase site and the SARS-CoV, MERS-CoV, and SARS-CoV-2 binding sites [14, 15], and a transmembrane C-terminal tail. ACE2 is a key player of the nonclassical RAS. Donoghue et al. [21] and Tipnis et al. [22] identified ACE2 from the complementary DNA library of heart failure and lymphoma patients in 2000 and inaugurated a new wave of studies of the real and expanded RAS.

ACE2, by cleaving the Pro7-Phe8 bond of Ang II, leads to the generation of the vasodilator and natriuretic peptide Ang1-7. ACE2 also cleaves Ang I into angiotensin 1-9 (Ang1-9) [20, 23], catabolizes other non-RAS peptides (apelin, kinins, and endorphins), and regulates the absorption of tryptophan in the intestine [24].

ACE2 is mainly located in the epithelial lining of the upper airways, in alveolar type II pneumocytes and pulmonary macrophages [25], as well as in the upper esophagus, colon, and surface enterocytes of the small intestine [26, 27]. Moreover, endothelial cells, testis, liver, kidney, and cardiac pericytes express ACE2 [28].

In the brain, ACE2 is expressed in astrocytes and astrocytic foot processes, pericytes and endothelial cells in the olfactory bulb, the hypothalamic nuclei, the midbrain substantia nigra, and the hindbrain pontine nucleus. Discrete neuronal groups express ACE2 in brainstem respiratory rhythm-generating centers (e.g., the pontine nucleus), in the arousal-related pontine reticular nucleus, and in the hippocampus [29].

Along with ACE2, three more enzymes play key roles in the nonclassical RAS [24, 30, 31] (Figure 1):

- ACE is a dicarboxypeptidase that notoriously cuts the Phe8-His9 bond of Ang I and generates Ang II in the ACE-Ang II-AT1R pathway. ACE has supporting roles also in the nonclassical RAS: it cleaves angiotensin 1-12 (Ang1-12) into Ang I, Ang1-9 into Ang1-7, and, finally, Ang1-7 into angiotensin 1-5 (Ang1-5) [20, 23]. Moreover, ACE catabolizes enkephalins, substance P, and luteinizing hormone-releasing hormone [24]. Most renal tubular cells and glomerular mesangial cells contain ACE. Finally, ACE is found in endothelial cells, especially in the blood vessels of the lung [20, 23, 24].
- Chymase is a serine endopeptidase found in the heart, liver, renal tubules, and mast cells. Chymase converts Ang I into Ang II in tissues the same as ACE in the vascular endothelium [24]. In areas of chronic inflammation, chymase also converts big endothelin-1 (big ET-1) into endothelin-1 (ET-1) [32] and releases transforming growth factor-*β* (TGF-*β*) through Ang II-dependent mechanisms [33]. 80% of Ang II synthesized in the blood vessel walls is dependent on chymase [34], but chymase inhibitors, unlike ACE inhibitors (ACEis), do not affect blood pressure and renin levels because ACE is located in endothelial cells and chymase in mast cells of the vascular adventitia of arterial vessel walls. Moreover, plasma contains serine endopeptidase (chymase) inhibitors [35]. Finally, chymase also cleaves Ang1-12 into Ang II
- Neprilysin (NEP) is a membrane-bound Zn-metalloendopeptidase also called atriopeptidase because neprilysin cleaves urodilatin, atrial, brain-derived, and C-type natriuretic peptides mostly in the kidneys, lung, brain, and heart [36]. NEP catabolizes opioid peptides, bradykinin, bombesin-like peptide, substance P, and adrenomedullin [37]. Inside pathways of nonclassical RAS, NEP cleaves Ang II into Ang1-5, Ang I into Ang1-7, and Ang1-7 into angiotensin 1-4 (Ang1-4) [20, 38]. This means that, based on the available precursor, NEP both generates and degrades Ang1-7

These enzymes, including ACE2, are anchored to the membranes of cells and orient their active sites on the extracellular cell surface to process substrates within the blood, glomerular filtrate, and interstitial fluids.

Ang1-7, the product of ACE2 action on Ang II, binds to cell membrane G protein-coupled receptors called Mas receptors (MasRs). MasR stimulation leads to enhanced phosphorylation of protein kinase B and nitric oxide production, to increased cell levels of cyclic GMP, and to increased production of prostaglandins G2, H2, and prostacyclins [20, 24, 38]. Natriuretic and vasodilator MasRs are ubiquitous but show the highest expression in the brain and testis [39].

The nonclassical RAS encompasses much more than this ACE2-Ang1-7-MasR axis. For instance, Ang1-7 may be transformed into the heptapeptide alamandine by an aspartate decarboxylase that converts Asp1 of Ang1-7 into Ala1. In turn, alamandine binds to the so-called Mas-related G protein-coupled receptor member D (MRGD), leads to vasodilatation, and has several health benefits owing to its antithrombogenic, anti-inflammatory, and antifibrotic characteristics [23, 40].

To sum up, Ang1-7 may be generated from Ang II through ACE2, from Ang I through NEP, and from Ang1-9 through ACE. This same heptapeptide may be metabolized to alamandine through aspartate decarboxylases or degraded to Ang1-5 through ACE or to Ang1-4 through NEP.

RAS peptidases behaviour is also versatile because their activity is strictly linked to the available substrate: monocarboxypeptidase ACE2 converts Ang I into Ang1-9 or Ang II into Ang1-7; dicarboxypeptidase ACE converts Ang I into Ang II or Ang1-7 into Ang1-5; NEP can remove three C-terminal amino acid residues from Ang I to form Ang1-7 or catabolizes Ang1-7 to form Ang1-4.

## 3. Links among Infectious Agents, a Complex Metabolic System, and an Inflammatory Cascade

Let us take *β*-coronaviruses as a paradigm of RAS involvement in viral infectious diseases.

These viruses express a glycoprotein called spike (S glycoprotein) on the viral coat. S glycoprotein contains two functional domains: an S1 ACE2-binding domain and an S2 domain necessary for fusion of the viral envelope and cell membranes. Host trypsin-like serine protease TMPRSS2 cuts between S1 and S2, and this process of spike priming is essential to allow viral entry into cells [26]. It is just the higher affinity of the SARS-CoV-2 S-protein for cell membrane ACE2 that explains why SARS-CoV-2 is far more infectious than SARS-CoV [7].

Beside TMPRSS2, two further serine proteases, furin and plasmin, may prime the SARS-CoV-2 S glycoprotein by cleaving it differently from TMPRSS2. High plasmin levels occur in cirrhotic patients because of increased activity of tissue-type plasminogen activator and decreased alpha 2-antiplasmin [41]. Therefore, the coexistence of liver cirrhosis aggravates the COVID-19 clinical syndrome [23]. As for furin, this serine protease of the subtilisin-like proprotein convertase family exerts priming effects on the receptor-binding domain (RBD) of different viruses: the human immunodeficiency virus (HIV), the influenza virus, the dengue fever virus, and several filoviruses, including the Ebola virus (EBOV). Therefore, inhibitors of furin are under consideration as therapeutic agents [18].

Following S glycoprotein priming, however determined, clathrin-dependent endocytosis of the parent virions, tightly bound to ACE2, into cells occurs and infection starts [23].

**Figure 2:**
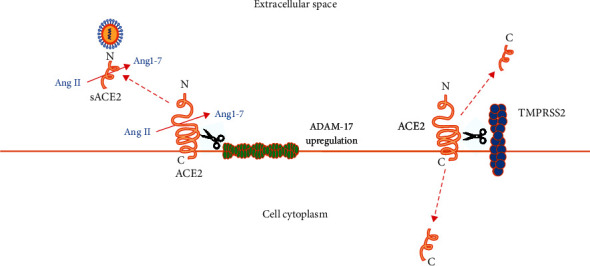
Left-hand side of the picture: soluble ACE2 (sACE2), obtained through the action of ADAM-17 on cellular ACE2, is the complete N-terminal ectodomain of the enzyme, which is still able to bind SARS-CoV-2 and convert Ang II into Ang1-7 in the extracellular space. Right-hand side of the picture: C-terminal ACE2 fragments of 13 kDa results from TMPRSS2 processing of cellular ACE2. Arginine and lysine residues within ACE2 amino acids 697 to 716 are essential for ACE2 cleavage by TMPRSS2; ADAM-17 requires arginine and lysine residues within ACE2 amino acids 652 to 659 for cleavage.

**Figure 3:**
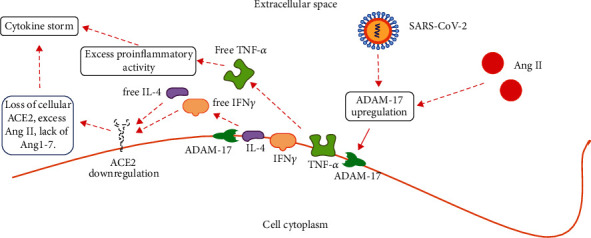
SARS-CoV-2 causes cell membrane ACE2 depletion through cellular internalization and subsequent proteasomal degradation once the enzyme is bound to infecting virions, but also through ADAM-17 upregulation. Ang II contributes to ADAM-17 upregulation. Upregulated ADAM-17 causes shedding of ACE2, interleukin-4, IFN*γ*, and TNF-*α* into extracellular fluids. In turn, free interleukin-4 and IFN*γ* further downregulate ACE2 cellular expression, while free TNF-*α* starts the inflammatory cascade.

In patients with COVID-19, internalization and the subsequent proteasomal degradation of ACE2 reduce its availability and function on the cell surface. Moreover, unknown SARS-CoV-2 components induce gene expression of a disintegrin and metalloproteinase domain-17 (ADAM-17) [23]. ADAM-17 is a membrane-bound zinc endopeptidase related to a family of enzymes known as sheddases or secretases. Sheddase ADAM-17 releases anchored ACE2 (Figure 2), interleukin-4 (IL-4), interferon *γ* (IFN*γ*), TNF-*α*, and IL-1 from human cell membranes. In turn, free IL-4 and TNF-*α* further downregulate the expression of membrane-bound ACE2 (Figure 3). TMPRSS2, beyond priming SARS-CoV-2 spikes, also cleaves ACE2 and competes with ADAM-17 for ACE2 extracellular shedding [26] (Figure 2). Inhibition of ADAM-17 with the vitamin D analog paricalcitol [42] or through knockdown by short interfering RNA (siRNA) successfully suppresses cellular infection by SARS-CoV-2 in vitro [43].

The degree of ACE2 extracellular shedding by ADAM-17 in the form of soluble ACE2 (sACE2, the complete N-terminal ectodomain of the enzyme) revealed a significant association with acute myocardial infarction and circulatory shock in COVID-19 patients, because elevation in sACE2 reflects cellular depletion of ACE2 and diminished tissue protection against Ang II-mediated microvascular damage [43].

The release of TNF-*α* through ADAM-17 is essential to COVID-19 pathogenesis (Figure 3). Two related forms of this cytokine have been described: a soluble form, sTNF-*α*, and a membrane-bound form, mTNF-*α*. There are two membrane receptors which sTNF-*α* interacts with to generate its pathological responses: these cell surface receptors are called TNFR-1 and TNFR-2 [44]. TNF-*α* starts the cytokine release required to initiate the inflammatory response in the lungs [45], which is characterized by increased vascular permeability and accumulation of pulmonary inflammatory fluid [46]. TNF-*α* also leaves the primary site of infection, reaches different target tissues as a systemic messenger of inflammation, and causes a generalized systemic inflammatory response [47]. TNF-*α* is thought to be the key mediator of the systemic inflammatory response syndrome.

Since ACE2 metabolizes also des-arginine bradykinin (des-Arg1-BK), this peptide, when ACE2 is lacking, becomes a promoter of pulmonary inflammation via stimulation of bradykinin B1 receptors in lung endothelial cells [48].

Finally, lack of ACE2 promotes considerable oxidative stress. The ACE2/Ang1-7/Mas axis counterregulates oxidative damage in the vascular system by reducing reactive oxygen species (ROS) production by NADPH-oxidase (NOX) [49]. Infusion of recombinant human ACE2 (rhACE2) in patients with pulmonary hypertension is associated with increased plasma levels of superoxide dismutase 2 (SOD2) and reduced oxidative stress [50] (Table 1).

Neutrophils recruited in the lungs of patients with atypical viral pneumonia are a key source of matrix metalloproteinase 9 (MMP-9), which is a matrixin, a class of enzymes that belong to the zinc-metalloproteinase family involved in the degradation of the extracellular matrix [47]. ROS, which are released after the binding of Ang II to AT1Rs followed by NOX activation, increase the collagenolytic activity of MMP-9 and extracellular matrix degradation [51] (Table 1). TNF-*α* and MMP-9 are interrelated, and both stimulate the release of the other during inflammation [47].

Loss of ACE2 leads to a general shift of the RAS to a higher Ang II and lower Ang1-7 tone. Ang1-7 binds to the C-terminal domain of ACE and reduces Ang II generation. Conversely, Ang II can trigger the phosphorylation of the microtubule-associated protein (MAP) kinases and the activation of extracellular signal-regulated kinase (ERK) 1 and 2 pathways, thus reducing ACE2 cell expression and upregulating transcription of profibrogenic genes such as TGF-*β*, plasminogen activator inhibitor-1, fibrillar collagens, and fibronectin [23] (Table 1). Therefore, ACE inhibitors (ACEis) and AT1R blockers (ARBs) increase ACE2 activity, as shown experimentally [15]. The conundrum is that ACEis and ARBs, through increased ACE2 expression and reduced Ang II function, may improve outcomes in patients with ARDS but may theoretically increase susceptibility to SARS-CoV-2 infection through increased expression of the viral receptor [15]. Recent meta-analyses demonstrate that RAS inhibitors are associated with a better prognosis in hypertensive patients with COVID-19 [52] and should not be discontinued in these subjects [53]. Inhibition of Ang1-7 clearance by ACEis may in part explain this finding (Figure 1). Moreover, reduced Ang II production decreases the enzymatic activity of ADAM-17 and, indirectly, the circulating levels of TNF-*α* [47] (Table 1). Finally, a study from the United Kingdom that included more than 15,000 patients with COVID-19 and 70,000 controls showed that ACEIs and ARBs did not increase but actually decreased the risk of SARS-CoV-2 infection in treated hypertensive subjects [54].

**Figure 4:**
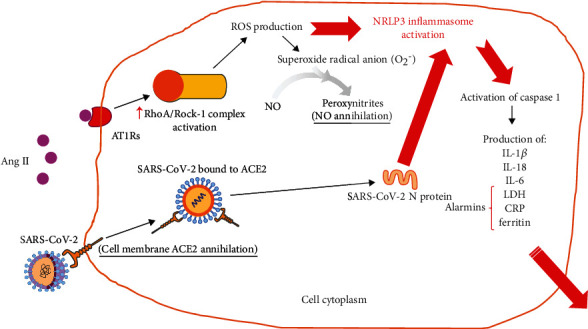
Schematic depiction of intracellular NRLP3 inflammasome activation due to SARS-CoV-2 and Ang II excess, and its consequences. Internalized ACE2 bound to virions undergoes proteasomal degradation. Intracellular SARS-CoV-2 N protein leads to direct NRLP3 inflammasome activation.

Ang II binding to AT1Rs prompts RhoA/Rock-1 complex activation, which leads to NOX-dependent ROS production [55] (Table 1 and Figure 4). In turn, ROS lessen nitric oxide (NO) bioavailability with subsequent endothelial dysfunction [47] (Figure 4). Conversely, activation of the ACE2/Ang1-7/Mas axis in the vascular endothelium increases production of vasodilators NO and prostacyclin by vascular smooth muscle cells [56]. The transcription level of the inflammatory inducer NF-*κ*B highly increases upon stimulation of AT1Rs by Ang II [55]: AT1R stimulation leads to phosphorylation of serine residues on I*κ*B*α* by the I*κ*B kinase. This results in the degradation of I*κ*B*α*, a natural inhibitor of NF-*κ*B. This way, NF-*κ*B is free to move into the cell nucleus and interact with proinflammatory target genes (e.g., the TNF-*α* gene), leading to their transcription [24, 47].

The COVID-19 prognosis is related to age and sex. Nonetheless, the expression of ACE2 decreases with increasing age: the ACE2 expression is higher in young people than in elderly individuals and higher in females than in males [57, 58]. This pattern does not match the characteristics of severely ill COVID-19 patients, being mostly elderly males. These findings underline that the patients endowed with a more developed “anti-inflammatory” ACE2 system do better, once infected by SARS-CoV-2, despite ACE2 being the cell receptor of the virus. In other words, patients at increased susceptibility to COVID-19 complications may have reduced baseline ACE2 [59].

The above findings do not seem to support the newly proposed use of ursodeoxycholate (UDCA) as the primary prophylaxis of COVID-19 [60]. In fact, UDCA and phytosteroid z-guggulsterone have been proposed to decrease the risk of the SARS-CoV-2 primary infection because both drugs, by inhibiting the activity of the bile acid receptor and transcription factor FXR, decrease ACE2 expression on the plasma membrane of cells in the gastrointestinal tract, pharynx, bronchi, lungs, and systemic circulation. And this treatment, at least in human organoids and perfused human lungs ex vivo, seems to reduce the rate of infection by SARS-CoV-2. Admittedly, the authors of such studies do not hide the fact that depriving the ACE2 body pool with UDCA to prevent dissemination of SARS-CoV-2 infection (secondary prophylaxis) might pose risks for patients already infected. In effect, the FXR (and, therefore, ACE2) activator obeticholic acid, despite the upregulation of ACE2, may paradoxically reduce COVID-19 disease severity and ameliorate cholestasis [60].

## 4. RAS and Inflammasome Activation (Figure 4)

Inflammasomes are multiprotein cytosolic complexes that assemble in monocytes, macrophages, and barrier epithelial cells in response to pathogen- or damage-associated molecular patterns. Upon activation, inflammasome sensors oligomerize to form mature inflammasomes, within which caspase 1 is activated. In turn, a proinflammatory lytic cell death called pyroptosis may occur [61], because caspase 1 processes pro-interleukin-1*β* (pro-IL-1*β*) and pro-interleukin-18 (pro-*Ι*L-18) into their active forms, which are also released into extracellular fluids along with alarmins such as lactate dehydrogenase (LDH).

NLRP3, a member of the nucleotide-binding domain- and leucine-rich repeat-containing protein (NLRP) family, responds to an array of insults to the cell that cause cytosolic K^+^ efflux, Ca^2+^ cytosolic influx, or release of mitochondrial ROS [61, 62].

Inflammasome activation in COVID-19 is testified by studies that revealed serum LDH concentration as the strongest predictor of severe disease [11]. Inflammasome activation is also accompanied by a release of serum markers of inflammation such as the interleukin-6-inducible C-reactive protein and ferritin, both associated with a severe prognosis of COVID-19 [63, 64]. Finally, interleukin-18 is a highly predictive biomarker of death by COVID-19 [65], and measurements in bronchoalveolar lavage fluid (BALF) showed a significant increase in the interleukin-1*β* (IL-1*β*) level in patients with moderate to severe COVID-19 [66]. Postmortem histological sections from lung parenchyma also showed broadly elevated staining of IL-1*β* compared with control sections [67].

Another strong indicator of inflammasome involvement in COVID-19 has been conclusively demonstrated: the N protein of SARS-CoV-2 directly induces NRLP3 inflammasome activation [11].

Generally, both NOX-derived ROS and mitochondrial ROS contribute to NLRP3 inflammasome activation. For instance, Ang II induces liver fibrosis in chronic liver diseases by NLRP3 inflammasome activation through a NADPH-oxidase 4- (NOX4-) and H_2_O_2_-dependent mechanism. Conversely, in hepatic stellate cells, Ang1-7 inhibits the Ang II-induced activation of the NLRP3 inflammasome [68]. In addition, in vivo activation of the NLRP3 inflammasome parallels an increase in the AT1R protein level and ROS production, as shown in human oral fibrosis tissues. Once again, Ang1-7 improves arecoline-induced rat oral submucosal fibrosis through reduction of protein levels of NOX4 and the NLRP3 inflammasome [69] (Table 1).

## 5. Restoration of ACE2 Function as a Suitable Clinical Answer

Several studies have shown that Ang1-7, through stimulation of MasRs, reduces the release of proinflammatory TNF-*α*, interleukin-6, and TGF-*β*, which trigger cell apoptosis and necrosis followed by tissue fibrosis. For instance, experimental liver fibrosis is aggravated by MasR antagonists [70] and relieved by recombinant ACE2 [71]. Thus, it appears that having excess ACE2 is beneficial to the patient with acute or chronic inflammation.

RAS imbalance, i.e., increase in ACE and decrease in ACE2 activities, contributes to ARDS development. In the rat model of ARDS caused by lipopolysaccharides, ACE activity and the Ang II content of the bronchoalveolar lavage fluid increase significantly, while the corresponding expression of ACE2 and Ang1-7 decreases [72]. Another study showed that in the mouse model of ARDS caused by bleomycin, the ACE2 gene-deficient mice had the most severe symptoms, which were relieved by treatment with rhACE2. ARDS symptoms were also relieved when applying AT1R blockers (ARBs), and the lung injury in mice with AT1R deletion was less severe [73].

During the outbreak of SARS in 2002, many patients developed ARDS and died. In those patients, the Ang II plasma levels increased significantly, and the expression of ACE2 was downregulated, resulting in lung injuries [74]. In human trials, patients with ARDS of different etiologies treated with i.v. rhACE2 showed reduction in Ang II and increase in Ang1-7 levels, although rhACE2 failed to improve significantly the clinical indicators of ARDS but, at least, was well tolerated [75].

Based on the above evidence, it can be concluded that ACE2 has a protective effect on lung injury and that ACE2 downregulation aggravates lung damage but that rhACE2 exogenous administration does not improve significantly the prognosis of patients with ARDS [73].

Finally, ACE2 exerts potent antithrombotic, anti-inflammatory, and antioxidant effects through cleavage of Ang II into the beneficial Ang1-7 [43]. Loss of ACE2 increases monocyte-endothelial adhesion, macrophage activation, vascular permeability, and oxidative stress, which exacerbate endothelial dysfunction [56]. Moreover, interleukin-6, whose secretion is blunted by Ang1-7, promotes the release of *α*-defensin, a prothrombotic peptide released by human neutrophils [76]. Not unexpectedly, the COVID-19 clinical profile includes coagulopathy, thrombosis, and endotheliitis in the microvasculature [77].

Once verified that atypical viral pneumonia and most experimental models of ARDS are indeed characterized by downregulation or, in the case of *β*-coronaviruses, actual annihilation of ACE2 everywhere this enzyme is located (i.e., lungs, heart, brain, blood vessels, kidney, and liver), how does the ACE/ACE2 imbalance manifest itself?

One study showed very low plasma levels of Ang1-7 and its catabolite Ang1-5 and even of Ang I, in patients with COVID-19 vs. healthy controls. Unexpectedly, lower, not higher, serum levels of Ang II were found in those same COVID-19 patients as compared with matched healthy controls [78]. Of course, serum levels of Ang II may not represent the actual tissue levels of the octapeptide. Moreover, it has to be stressed that the main degradative pathway of Ang II is not effected by ACE2, whose function is clearly damaged by SARS-CoV-2 infection, but through the sequential actions of aminopeptidases A and N, which lead to the production of angiotensin 2-8 (Ang2-8) and then angiotensin 3-8 (Ang3-8) [23] (Figure 1). This means that lack of ACE2, by itself, may not necessarily increase the serum levels of Ang II. The uncertainties about relative excess or lack of RAS peptides recently arrived at such a point that infusion of Ang II itself was taken into consideration to treat the most severe patients with COVID-19: of course, the results of such attempts were disappointing [79].

Another study compared prolonged viral shedders (nasopharyngeal positive SARS-CoV-2 PCR ≥ 10 days from the first consultation) to short viral shedders (nasopharyngeal positive SARS-CoV-2 PCR < 10 days from the first consultation) and showed that Ang II serum concentrations were significantly higher in prolonged viral shedders than in healthy controls or short viral shedders [80].

The last word came from a large cohort of Chinese patients with COVID-19, where plasma Ang II levels were found increased, and these hormonal levels correlated with the viral load in the bronchoalveolar lavage fluid [81].

Therefore, due to the ACE/ACE2 imbalance occurring in atypical viral pneumonia, we do find definitely increased Ang II and dramatically decreased Ang1-7 levels mostly in tissues but also in extracellular fluids.

Atypical viral pneumonia, even when unrelated to the *β*-coronavirus infection, is frequently characterized by a lack of tissue ACE2. In summary, severe inflammatory reactions with a local and systemic release of TNF-*α* and interleukin-4 or activation of ADAM-17 lead to ACE2 downregulation and extracellular shedding (Figure 3). In turn, lack of cell membrane ACE2 brings about ACE/ACE2 imbalance, increased Ang II, and decreased Ang1-7 tissue levels, fuelling further local inflammation and oxidative stress (Figure 4).

Not unexpectedly, patients with acute lung injury due to avian influenza A H7N9 and H5N1 viruses or hRSV show significant downregulation of lung ACE2 and elevated Ang II serum levels [16, 17]. In these cases, since the cell receptor of hRSV and influenza viruses is not ACE2 and ACE2 therefore does not get internalized into cells attached to these infecting agents, it is the intrinsic mechanism of inflammation ignited by these viral agents that leads to the secondary depletion of tissue ACE2, through TNF-*α*, interleukin-4, or ADAM-17 activation. A further proof of this mechanism comes from the study of cell infection by hepatitis C virus or cytomegalovirus: these two viral agents do not decrease but actually increase cell expression of ACE2 in in vitro infected epithelial cells but, nonetheless, cause significant local and systemic inflammation [82, 83].

## 6. New Treatment Strategies Provided by Nonclassical RAS Functioning

It is reasonable that if we could in some way replace ACE2 that is primarily or secondarily lost in infected tissues, we would potentially decrease the extent of systemic inflammation and local damage in patients with atypical viral pneumonia of different etiologies [84]. Restoring the Ang1-7 content in tissues despite inadequate ACE2 function might be an alternative novel strategy. Notably, the latter metabolic approach would be eligible in every clinical case of COVID-19, irrespective of the genetic variant of SARS-CoV-2 involved, and in ALI due to hRSV and avian influenza viruses.

In COVID-19, it was initially thought that rhACE2 systemic administration would either prevent viral spread or inhibit the secretion of proinflammatory mediators [85].

Endogenous plasma levels of soluble ACE2 are almost undetectable and unable to sequester SARS-CoV-2 in the circulation to prevent viral dissemination. Six months after the onset of the pandemic, the first COVID-19 patient treated with intravenous rhACE2 was described [86, 87]. Nine days after the onset of COVID-19 symptoms, the patient received rhACE2 twice daily for seven days by intravenous infusion: a marked reduction in serum Ang II levels with concomitant increases in Ang1-7, Ang1-9, and their by-product Ang1-5 was promptly observed. The copy number of SARS-CoV-2 decreased dramatically, as well as the systemic levels of cytokines interleukin-6 and interleukin-8. The patient survived. This initial enthusiasm raised by i.v. rhACE2 was rapidly blunted: a clinical trial of infused rhACE2 was proposed and subsequently withdrawn in China because ACE2 infusion, decreasing Ang II and increasing Ang1-7 systemic levels too much, caused considerable arterial hypotension and cardiovascular side effects in patients with advanced stages of COVID-19 [7, 15].

Since soluble ACE2 may at least act as bait to neutralize the spike protein on the surface of SARS-CoV-2, a fusion protein containing a modified ACE2 enzyme with a low catalytic activity bound to the Fc region of IgG1 was produced. This compound showed a good binding affinity for the receptor-binding domain (RBD) of SARS-CoV and SARS-CoV-2 in infected mice [88].

Intranasal delivery of ACE2 has also been proposed. In this case, modified ACE2 molecules were administered by an inhaler during the early phases of the COVID-19 infection. This approach should reduce the number of virions that infect the nasal mucosa. Consequently, there should be fewer virions that can reach the lungs, and by this route, a portion of drugs could also reach the brain from the nasal cavity [89, 90]. This way, adverse cardiovascular events should not occur, even if some of the drug enters the circulation [90].

A further strategy implies a soluble ACE2 variant fused with an albumin-binding domain (ABD) (ACE2–1-618-DDC-ABD). This drug was administered intranasally and intraperitoneally to mice before and after intranasal inoculation of SARS-CoV-2: untreated animals died by day 7 due to pulmonary alveolar hemorrhage with mononuclear infiltrates; in contrast, almost all mice infected with a lethal dose of SARS-CoV-2 that received ACE2–1-618-DDC-ABD survived [91].

ACE2-derived peptides potentially neutralizing the RBD of the SARS-CoV-2 S1 domain have also been identified. It was shown that amino acid sequences placed at the 21-57 and 351-357 positions of the N-terminal helix of ACE2 allow the interaction with SARS-CoV-2 RBD [87, 92].

Finally, extracellular vesicles that express ACE2 (evACE2) were isolated from the plasma of patients with COVID-19. evACE2 neutralizes SARS-CoV-2 infection by competing with cellular ACE2 and protects human ACE2 transgenic mice from SARS-CoV-2-induced lung injury [93].

When it comes to the atypical viral pneumonia of different etiologies but characterized by tissue ACE2 loss, a completely different approach may be hypothesized. Provided by the functional flexibility of RAS peptidases, this approach consists in replacing defective Ang1-7 production without resorting to systemic administration of exogenous ACE2, which otherwise would cause Ang1-7 production inside blood vessels, arterial hypotension, and cardiovascular complications.

Flexibility of RAS means that the same component can produce opposite physiological effects through different pathways (e.g., Ang II may be antinatriuretic when bound to AT1Rs or natriuretic when bound to Ang II type 2 receptors [AT2Rs]), and different components can have the same physiological effect by different pathways (e.g., Ang1-7 is produced from Ang II by ACE2 or from Ang I by NEP). Moreover, when a branch of RAS is blocked, synthesis of angiotensins may find its way along another path of the RAS metabolism that is still practicable (Figure 1). For instance, when ACEis are used, a quota of aldosterone is still released by the adrenals due to Ang II being newly produced by chymase and cathepsin G [94]. Another example is as follows: when ACE2 is blocked by specific metallopeptidase inhibitors, NEP starts cleaving Ang I into Ang1-7, provided enough substrate for the reaction is available [95]. In addition, it was shown that Ang1-7 is the most common metabolite of Ang I in certain areas of the brain (i.e., the hippocampus) where thimet oligopeptidase and prolyl endopeptidase (PEP) are the enzymes involved in the generation of the heptapeptide [96]. Finally, even carboxypeptidase A and prolyl carboxypeptidase (PCP) may generate Ang1-7 from Ang II [56].

Understanding this flexibility of nonclassical RAS suggests the following strategy.

Captopril can reduce pulmonary hypertension, delay the progression of ARDS, and protect lung vascular endothelial cells in rat models of oleic acid-induced ARDS or endotoxin-induced lung injury [97], and it was shown that even ARBs have a therapeutic effect in human ARDS [77]. Mostly because ACEis inhibit clearance of Ang1-7 into inactive Ang1-5 (Figure 1), while ARBs do not, ACEis should be administered to normo-hypertensive patients affected by atypical viral pneumonia due to ACE2 loss; what is more, concurrent i.v. infusion of Ang I, which does show very low serum concentrations at least in COVID-19 patients [70], should be associated. This decapeptide is not hypertensive, provided ACE is inhibited, and is the substrate for Ang1-7 synthesis by NEP in the lung. This strategy would possibly lead to increased tissue production of Ang1-7 by NEP, without significant spillover of this hypotensive heptapeptide into the systemic circulation.

Previous experiments seem to support this atypical strategy, which involves ACEis and Ang I administration at the same time.

Firstly, when the rat cirrhotic liver was perfused with metallopeptidase inhibitors, ACE2 inhibition dramatically increased hepatic Ang1-7 production from Ang I, an effect abolished by NEP inhibitors. This means that ACE2 inhibition unleashed Ang I cleavage into Ang1-7 by hepatic NEP [95]. The authors of this review had previously described the overexpression of NEP inside the cirrhotic liver [37].

Secondly, since NEP mRNA and NEP-immunoreactive material are largely detected in the bronchial epithelial cells, submucosal glands, smooth muscle, and endothelium [98], NEP could replace inadequate ACE2 function at least in the lung if its occasional substrate Ang I was freely available.

Thirdly, in a recent review, it was postulated that increasing NEP activity might mitigate by itself COVID-19 severity [99]. NEP seems to play a protective role in the lung since, in the experimental model of mice with acute lung injury, a significant decrease in NEP enzymatic activity occurs. This leads to defective tachykinin clearance and Ang1-7 synthesis and uncontrolled lung inflammation [100].

Finally, it is important to remember that NEP may also degrade Ang1-7, but this peptidase is reported to be involved in the catabolism of Ang1-7 into Ang1-4 within tissues other than the lung, mostly in the renal cortex [101, 102]. Indeed, the major enzyme responsible for Ang1-7 catabolism (into Ang1-5) in the pulmonary tissue is ACE [101].

A strategy pursued against diseases caused by genus *β*-coronaviruses is the attempt to modify the host cell membrane ACE2, the viral receptor.

After the SARS-CoV outbreak of 2002, metallopeptidase inhibitors (e.g., MLN-4760) were produced to alter the physical conformation of ACE2, thereby preventing the coronavirus from binding to cell membranes. Unfortunately, those drugs led to further inhibition of ACE2-dependent Ang1-7 production [103].

Recently, novel pyrazolone-based compounds derived from edaravone, a vasodilator of brain and coronary arteries, were designed as potential inhibitors that would interrupt the interaction between the SARS-CoV-2 S protein and the host cell receptor (ACE2). Notably, these new molecules do not alter the structural integrity of cell membrane ACE2, prevent attachment of the coronavirus spike to its receptor, and may not inhibit ACE2-driven production of Ang1-7 [104].

Serine protease inhibitors might prevent the host serine protease TMPRSS2 from priming the spike (S glycoprotein) of the SARS-CoV-2 viral coat prior to viral entry into human cells. With this aim, i.v. serine protease inhibitors camostat mesylate and nafamostat mesylate were administered to symptomatic patients with confirmed COVID-19 infection. In two different randomized clinical trials, camostat mesylate did not affect the time to clinical improvement or mortality, without significant adverse events [105, 106]. In a retrospective clinical study, nafamostat mesylate was ineffective against COVID-19 and, on top of this, frequently caused hyperkalemia due to unwanted inhibition of amiloride-sensitive sodium channels in the kidney [107].

These discouraging results must be balanced by the knowledge that 80% of Ang II-forming activity in kidney, heart, and blood vessels is dependent on another human serine protease: chymase [34] (Figure 1). Moreover, unlike ACE inhibitors, chymase inhibitors do not lower blood pressure because chymase is found in mast cells of the vascular adventitia [35]. Both chymase and TMPRSS2 are trypsin-like serine proteases belonging to family F1 and subfamily A of serine proteases, according to the MEROPS peptidase database. Therefore, the chymase inhibitor SF2809E would warrant consideration in COVID-19 because chymase is a ubiquitous serine protease quite like TMPRSS2, it is a source of detrimental peptides Ang II and ET-1 in human tissues and not in the systemic circulation [108], and SF2809E might also inhibit TMPRSS2. Moreover, SF2809E does not cause hyperkalemia and relieves sodium retention in a model of experimental liver cirrhosis with ascites [108]. It is not known whether chymase inhibitors affect cellular ACE2 expression as ACEis do.

Finally, serine protease (chymase) inhibitors circulate freely in the blood of otherwise healthy humans. These physiological serine protease inhibitors (serpins and *α*1-antitrypsin) have potent anti-inflammatory effects [109].

Therefore, efforts should be made to assess the ability of serpins, *α*1-antitrypsin, and, mostly, chymase inhibitors to block the host serine protease TMPRSS2 [26, 35].

## 7. Conclusions

Local and systemic loss of ACE2 is a key trigger of severe inflammatory syndromes caused by genus *β*-coronaviruses, avian influenza viruses, and the human respiratory syncytial virus and of almost every case of ARDS. It is known that attempts at restoring the ACE2 body content in these cases through systemic administration of this peptidase may lead to excessive intravascular production of the vasodilator Ang1-7 and intolerable side effects. The way nonclassical RAS works provides suggestions for restoring tissue Ang1-7 levels, the true target of therapies, without resorting to ACE2 administration.

In general, when key mechanisms of inflammation are recruited, i.e., ADAM-17 releasing soluble ACE2 from cell membranes and, mostly, TNF-*α* downregulating cell membrane ACE2, the strategy of replacing the Ang1-7 tissue production through ACEis, NEP, and its substrate Ang I, as we illustrate in this paper, might be of some use and is worth being evaluated through trials in human patients, not only in COVID-19 but also in acute lung injury due to hRSV and avian influenza viruses.

Finally, attempts at inhibiting host serine proteases that prime coronaviruses prior to cell infection, albeit initially discouraging, must not be abandoned. Instead, this effort should be renewed employing serine proteinase inhibitors capable of blocking both nonclassical RAS serine peptidase chymase (the main source of Ang II in tissues and vessel walls) and hopefully even serine peptidase TMPRSS2. Once again, clinical trials in humans are warranted.

## Figures and Tables

**Table 1:** Opposing effects of Ang II and Ang1-7.

*Ang II leads to:*
NADPH-oxidase-mediated reactive oxygen species production
Increased collagenolytic activity of MMP-9
Reduced ACE2 cell expression
Induction of the enzymatic activity of ADAM-17, leading to increased circulating levels of TNF-*α*
NLRP3 inflammasome activation
High transcription of NF-*κ*B leading to production of proinflammatory cytokines

*Ang1-7 leads to:*
Increased plasma levels of superoxide dismutase 2 and reduced oxidative stress
Reduced ACE function
Increased production of the vasodilator NO, prostaglandins H2 and G2, and prostacyclin
Inhibition of NADPH-oxidase-mediated reactive oxygen species production
Inhibition of NLRP3 inflammasome activation

## Data Availability

This review includes data from all the cited references. This is not a clinical trial.

## Abbreviations

ABD: Albumin-binding domain
ACE: Angiotensin-converting enzyme
ACE2: Angiotensin-converting enzyme type 2
ADAM-17: A disintegrin and metalloproteinase domain-17
ACEi: Angiotensin-converting enzyme inhibitor
ALI: Acute lung injury
Ang I: Angiotensin I
Ang II: Angiotensin II
Ang1-4: Angiotensin 1-4
Ang1-5: Angiotensin 1-5
Ang1-7: Angiotensin 1-7
Ang1-9: Angiotensin 1-9
Ang1-12: Angiotensin 1-12
Ang2-8: Angiotensin 2-8
Ang3-8: Angiotensin 3-8
ARB: Angiotensin II type 1 receptor blocker
ARDS: Acute respiratory distress syndrome
AT1R: Angiotensin II type 1 receptor
AT2R: Angiotensin II type 2 receptor
Big ET-1: Big endothelin-1
BALF: Bronchoalveolar lavage fluid
COVID-19: Coronavirus disease of 2019
des-Arg1-BK: Des-arginine bradykinin
EBOV: Ebola virus
ERK: Extracellular signal-regulated kinase
ET-1: Endothelin-1
evACE2: Extracellular vesicles that express ACE2
HIV: Human immunodeficiency virus
hRSV: Human respiratory syncytial virus
IFN*γ*: Interferon *γ*
IL-1: Interleukin-1
IL-1*β*: Interleukin-1*β*
IL-4: Interleukin-4
LDH: Lactate dehydrogenase
MAP kinase: Microtubule-associated protein kinase
MasR: Mas receptor
MERS: Middle East respiratory syndrome
MERS-CoV: Middle East respiratory syndrome coronavirus
MMP-9: Matrix metalloproteinase 9
MRGD: Mas-related G protein-coupled receptor member D
mTNF-*α*: Membrane bound tumour necrosis factor-*α*
NEP: Neprilysin
NO: Nitric oxide
NOX: NADPH-oxidase
NOX4: NADPH-oxidase 4
NLRP: Nucleotide-binding domain- and leucine-rich repeat-containing protein
PCP: Prolyl carboxypeptidase
PEP: Prolyl endopeptidase
Pro-IL-1*β*: Pro-interleukin-1*β*
Pro-*Ι*L-18: Pro-interleukin-18
RAS: Renin-angiotensin system
RBD: Receptor-binding domain
rhACE2: Recombinant human ACE2
ROS: Reactive oxygen species
sACE2: Soluble ACE2
SARS: Severe acute respiratory syndrome
SARS-CoV: Severe acute respiratory syndrome coronavirus
SARS-CoV-2: Severe acute respiratory syndrome coronavirus 2
siRNA: Short interfering RNA
SOD2: Superoxide dismutase 2
sTNF-*α*: Soluble tumour necrosis factor-*α*
TGF-*β*: Transforming growth factor-*β*
TNF-*α*: Tumour necrosis factor-*α*
TNFR-1: Tumour necrosis factor-*α* receptor 1
TNFR-2: Tumour necrosis factor-*α* receptor 2
UDCA: Ursodeoxycholate.
